# Administration of Topical NorLeu^3^Angiotensin(1-7) Minimizes Fibrotic Corneal Healing in Stellate Wound: A 28-Day Study

**DOI:** 10.3390/ijms27083565

**Published:** 2026-04-16

**Authors:** Catherine Chester, Edgar Alejandro Moreno-Diaz, Weiyuan Hu, Brianna Chen, Maram Alshammari, Mark S. Humayun, Juan Carlos Martinez Camarillo, Stan G. Louie

**Affiliations:** 1 Titus Family Department of Clinical Pharmacy, Alfred E. Mann School of Pharmacy and Pharmaceutical Sciences, University of Southern California, Los Angeles, CA 90089, USA; 2 Roski Eye Institute, Keck School of Medicine, University of Southern California, Los Angeles, CA 90089, USAhumayun@med.usc.edu (M.S.H.);; 3 USC Ginsburg Institute for Biomedical Therapeutics, University of Southern California, Los Angeles, CA 90089, USA

**Keywords:** cornea, renin–angiotensin system, fibrosis, corneal haze, corneal repair, myofibroblast, MasR

## Abstract

Severe full-thickness corneal lacerations disrupt the tight cellular and extracellular matrix (ECM) organization required for corneal transparency. Following injury, an influx of transforming growth factor beta (TGFβ) into the corneal stroma signals the formation of haze-inducing myofibroblasts, resulting in excessive stromal remodeling and corneal haze. We hypothesized that MasR activation using NorLeu^3^Angiotensin (1-7) (NLE) engages the pro-resolving arm of the renin–angiotensin system (RAS) to minimize fibrotic corneal repair. In this study, 6 mm stellate-shaped, full-thickness corneal lacerations were induced in New Zealand Black (NZB) rabbits and treated with topical vehicle, or 0.1%, 0.3%, or 0.45% NLE. Corneal healing was evaluated using noninvasive corneal imaging, histology, and the gene expression of RAS- and fibrosis-related targets (MasR, AT1R, TGFβR1). Corneal imaging revealed significantly decreased corneal haze (*p* < 0.05) and increased keratocyte density with 0.1% NLE treatment (*p* < 0.05). Immunofluorescence showed significantly reduced α-smooth muscle actin (αSMA), indicating decreased myofibroblast formation (*p* < 0.05). Additionally, 0.1% NLE reduced stromal TGFβR1, suggesting that NLE mediates its activity by disrupting the TGFβ/TGFβR axis. MasR and AT1R gene expression were downregulated, which contributes to a reduction in fibrosis. Collectively, these findings suggest that the NLE activation of MasR modulates RAS and TGFβ/TGFβR signaling to reduce myofibroblast activity and fibrosis following severe corneal trauma.

## 1. Introduction

The cornea is the transparent layer on the anterior surface of the eye that serves as a protective barrier against mechanical and infectious insults. It also provides ocular refractive power that is critical for visual acuity [1]. Epithelial, stromal, and endothelial cells found in the cornea are carefully aligned to focus light onto retinal photoreceptors [2]. This highly organized structure allows light to pass through the cornea without scattering, which is the basis of corneal transparency.

When the cornea is injured, the trajectory of corneal wound repair is dictated by the wound size, depth, and duration of transforming growth factor beta (TGFβ) signaling. Small or partial-thickness wounds typically re-epithelialize within several days, rapidly restoring the epithelial basement membrane (EBM) and restricting stromal exposure to pro-fibrotic mediators [3]. In contrast, more extensive or full-thickness wounds disrupt both EBM and Descemet’s membrane and prolong stromal exposure to TGFβ1/β2, which drives sustained myofibroblast differentiation and extracellular matrix (ECM) disorganization [4,5,6]. Myofibroblasts, identified by the expression of α-smooth muscle actin (αSMA), are formed from stromal keratocytes that differentiate in response to TGFβ1/β2 released from damaged epithelial cells [7,8].

During corneal repair following injury, keratocytes repopulate and the stromal ECM is remodeled with type I/V collagen and keratan sulfate proteoglycans (lumican, keratocan) in an attempt to restore the lamellar spacing essential for corneal transparency [9]. In partial-thickness corneal injuries, myofibroblast formation is brief and self-limited, as EBM restoration blocks pro-fibrotic signaling [10]. Collagen lamellae realign, proteoglycan composition normalizes, and stromal transparency is restored rapidly. In contrast, in full-thickness wounds, disruption of the EBM and Descemet’s membrane may sustain TGFβ entry into the stroma, leading to persistent myofibroblast activity [11]. These myofibroblasts secrete excessive, randomly oriented ECM (e.g., fibronectin, collagen type III) and may suppress transparency-associated genes like keratocan, resulting in fibrosis and stromal haze [12,13,14,15,16].

Ultimately, the balance between fibrotic and clear corneal healing is determined by the intensity of TGFβ and TGFβ receptor (TGFβR) signaling, which is governed by the rate of epithelial basement and Descemet’s membrane restoration [11]. Severe open globe injuries that penetrate both of those layers, like those sustained in the combat theater, lead to permanent loss of visual acuity or even complete blindness [17,18]. Immediate treatment options are limited to supportive care, such as pain management and infection preventatives.

NorLeu^3^Angiotensin (1-7) (NLE) is a non-natural agonist of MasR developed by substituting the third position valine of angiotensin (1-7) with a norleucine [19]. The renin–angiotensin system (RAS) pathway, known for its role in regulating hemodynamics, has also been recognized for its role in ocular repair [20,21,22]. We have previously shown that the topical administration of NLE was able to promote clear corneal healing in a 2 mm full-thickness corneal wound model through MasR activation [23]. Here, we determined the optimal dosage of NLE in a severe corneal injury model, using a 6 mm stellate full-thickness corneal laceration to mimic traumatic injuries. Additionally, we interrogated the molecular mechanisms by which NLE exerts its therapeutic effect, which include the disruption of TGFβ/TGFβR signaling for myofibroblast formation and reducing the expression of angiotensin type 1 receptor (AT1R).

## 2. Results

### 2.1. Clinical Measurements

Mean intraocular pressure (IOP) reduced following surgery, as expected, and stabilized by Day 14 in all treatment groups (Figure S1). IOP measurements were not statistically different between treatment groups. Seidel’s test results indicated 37.5% (3/8), 62.5% (5/8), 57.1% (4/7), and 28.5% (2/7) of vehicle, 0.1%, 0.3%, and 0.45% NLE corneas sealed within 24 h, respectively. There were no statistical differences between the various treatment groups; however, 0.1% NLE trended towards accelerated wound closure.

### 2.2. Corneal Haze

**Figure 1:**
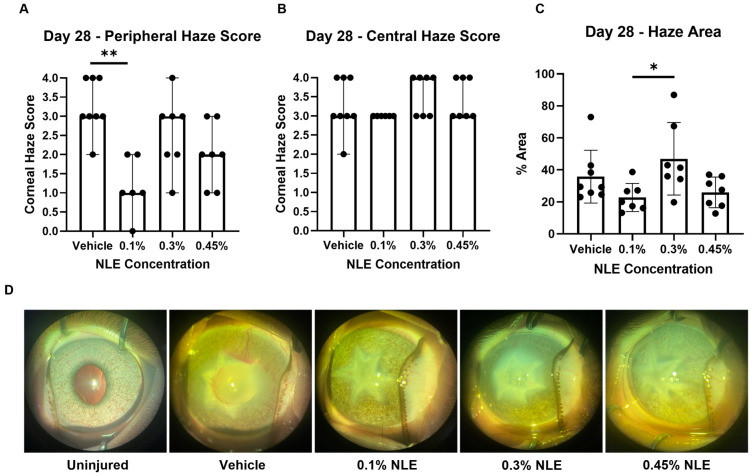
Evaluation of corneal haze. (**A**,**B**) Corneal haze scores indicate none (0), trace (0.5), minimal (1), mild (2), moderate (3), or severe (4). Median haze scores are plotted, and error bars represent range. (**C**) Area of corneal haze in pixels, normalized to corneal area in pixels, demonstrates reduced corneal haze with 0.1% NLE treatment compared with 0.3% NLE (*p* < 0.05). (**D**) Representative images of each treatment group on Day 28. ** *p* < 0.01. * *p* < 0.05.

Representative slit lamp images collected on Day 28 visualizing corneal haze are depicted in Figure 1D. Haze scores using three masked reviewers revealed less severe haze in the peripheral cornea than the central cornea, as expected (Figure 1A,B). Peripheral corneal haze significantly decreased with 0.1% NLE compared to vehicle (*p* < 0.01), while 0.3% and 0.45% NLE did not show statistical difference (Figure 1A). Central haze was unaffected, which is expected given the severity of the injury.

To corroborate the masked haze scoring results, the corneal haze area was quantified via ImageJ (version 1.54g). Corneal haze was not significantly different between treatment groups until Day 28 (Figure S2). Corneal haze area was significantly reduced with 0.1% NLE treatment (*p* < 0.05), supporting the results from the masked haze scoring (Figure 1C). These results show that 0.1% NLE treatment promotes minimal fibrotic corneal repair.

### 2.3. HRT-III Imaging of Corneal Architecture

**Figure 2:**
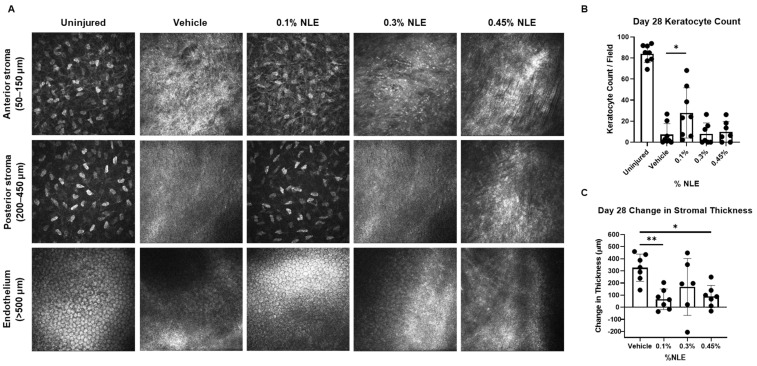
Corneal confocal microscopy imaging on Day 28. (**A**) Representative images (400 × 400 μm) from each treatment group are pictured for each layer and represent the keratocyte density throughout the stroma for treatment group. (**B**) Stromal keratocyte density was average from five HRT-III images from each stroma. Keratocyte levels are significantly higher in the 0.1% NLE treatment group (*p* < 0.05) than the vehicle. (**C**) The change in stromal thickness from Day 0 to 28 shows reduced corneal edema with 0.1% NLE (** *p* < 0.01) and 0.45% NLE (* *p* < 0.05).

Heidelberg Retinal Tomograph III (HRT-III) confocal microscopy was used to non-invasively monitor corneal morphological changes over time. Representative images taken on Day 28 of the stroma and endothelium are shown in Figure 2A. The healthy and uninjured corneal stroma shows highly organized keratocytes in the absence of a hazy extracellular matrix (Figure 2A). Only corneas treated with 0.1% NLE show markers of healthy corneal stromal architecture on Day 28, defined by consistent keratocyte distribution and the absence of disorganized collagen lamellae. These observations are consistent with the reduced corneal haze noted in the 0.1% NLE treatment group (Figure 1).

Uninjured, healthy corneal endothelial cells were identified by their hexagonal morphology and cobblestone arrangement (Figure 2A). The endothelial layer was located anywhere from 500 to 1000 µm below the anterior surface, depending on the extent of corneal edema. It was observed in the vehicle, 0.3% NLE, and 0.45% NLE-treated corneas that less than half (37.5%, 3/8; 42.9%, 3/7; 42.9%, 3/7, respectively) showed an endothelial layer on Day 28. Endothelial restoration following severe injury is critical to regulating fluid and nutrient content in the cornea. A healthy cobblestone layer was evident in most corneas (75%, 6/8) treated with 0.1% NLE on Day 28, indicating enhanced corneal restoration with 0.1% NLE treatment. It is possible that this phenomenon is secondary to improved wound repair in this treatment group, rather than a direct effect of NLE on the endothelium itself.

To further evaluate NLE’s effect on corneal architecture, we developed an artificial intelligence (AI) model using Biodock.ai that is capable of quantifying corneal keratocytes in HRT-III images. Keratocyte density was measured at each timepoint, though no significant differences were observed until Day 28 (Figure S3). Keratocyte density was significantly greater in 0.1% NLE-treated corneas than vehicle-treated corneas (*p* < 0.05), and was closer to uninjured/healthy levels than all other treatment groups, indicating greater wound resolution in the 0.1% NLE treatment group (Figure 2B).

The change in stromal thickness from Day 0 to Day 28 was assessed as a surrogate marker for corneal edema. Treatment with 0.1% NLE significantly reduced the change in stromal thickness when compared with the vehicle, indicating 0.1% NLE treatment reduces corneal edema (*p* < 0.01) (Figure 2C). Treatment with 0.45% NLE also reduced corneal edema compared to the vehicle (*p* < 0.05) (Figure 2C). High variability was noted in the 0.3% NLE treatment group.

### 2.4. Masson’s Trichrome Staining

**Figure 3:**
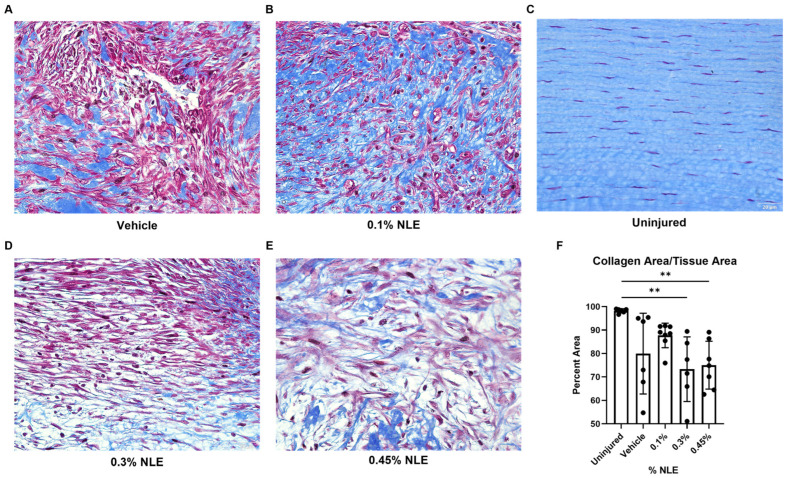
Corneal sections stained with Masson’s trichrome to visualize collagen formation (blue) in uninjured (**C**) and injured stroma (**A**,**B**,**D**,**E**). Images captured at 40× magnification. Collagen formation plotted as percent area of tissue stained blue (**F**). Collagen area significantly reduced following injury after treatment with 0.3% and 0.45% NLE. ** *p* < 0.01. Scale bar = 20 μm.

Masson’s trichrome staining was used to visualize post-injury collagen formation, as collagen alignment is key to corneal transparency and thus visual acuity. Healthy, uninjured corneas appear mostly blue, as collagen is the main structural component in the cornea (Figure 3C). Red-stained keratocytes lie parallel with collagen bundles in the uninjured corneas. In contrast, injured corneas show decreased blue collagen and increased red cytoplasm and keratin (Figure 3A,B,D,E). Additionally, the blue collagen fibrils in the injured corneas are disorganized. Following injury, collagen content is significantly decreased in the 0.3% and 0.45% NLE groups (Figure 3F) (*p* < 0.01). Treatment with 0.1% NLE significantly increases collagen reconstruction following injury, indicating more efficient wound repair in the 0.1% NLE treatment group.

### 2.5. Immunofluorescence Staining of αSMA

**Figure 4:**
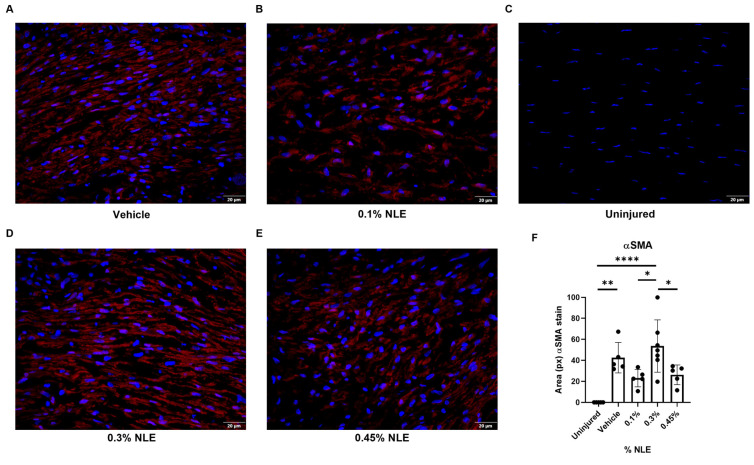
Immunofluorescence staining of αSMA (red) with DAPI (blue). Images captured at 40× magnification in uninjured stroma (**C**) and injured stroma (**A**,**B**,**D**,**E**). Area of αSMA stain is reduced with 0.1% and 0.45% NLE compared to 0.3% NLE (**F**). **** *p* < 0.0001; ** *p* < 0.01; * *p* < 0.05.

Persistent myofibroblast activity can exacerbate corneal haze; therefore, myofibroblast levels were quantified via immunofluorescence staining of αSMA. Uninjured corneas show no αSMA staining, as expected (Figure 4C). Following injury, αSMA is increased in the vehicle (*p* < 0.01) and 0.3% NLE (*p* < 0.0001) treatment groups (Figure 4F). The 0.1% NLE and 0.45% NLE treatment groups show decreased density of αSMA stain, indicating a reduction in myofibroblast activity that is consistent with the reduced corneal haze in these treatment groups (Figure 1A).

### 2.6. Immunofluorescence Staining of TGFβ1 and TGFβR1

**Figure 5:**
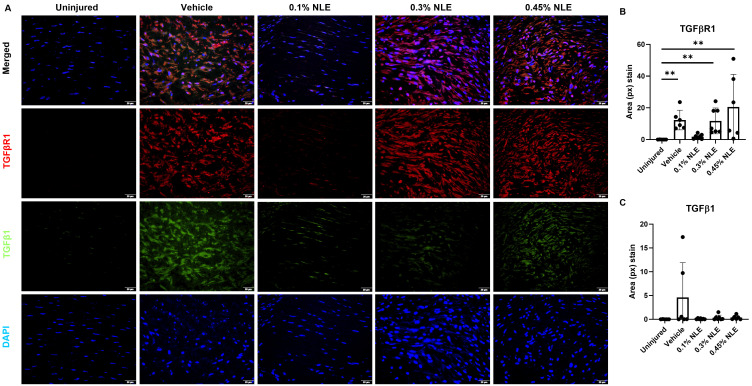
Immunofluorescence staining of TGFβR1 (red) and TGFβ1 (green) in the corneal stroma, counterstained with DAPI (blue). (**A**) Representative 40× images of uninjured and injured corneas. (**B**) Area of TGFβR1 is elevated in injured corneas treated with vehicle or 0.3% or 0.45% NLE. (**C**) TGFβ1 is somewhat elevated in vehicle-treated corneas. ** *p* < 0.01. Scale bar = 20 μm.

In the uninjured cornea, TGFβ1 and TGFβR1 are not observed in the stroma (Figure 5A). TGFβ1 levels in the stroma are elevated somewhat in vehicle-treated corneas, while NLE-treated corneas show little change in TGFβ1 levels compared to uninjured corneas, though these differences are not significant (Figure 5C). TGFβR1 is significantly increased in the stroma of vehicle- and 0.3% and 0.45% NLE-treated corneas compared to uninjured corneas (Figure 5B) (*p* < 0.01). In contrast, with 0.1% NLE treatment, TGFβR1 levels are unchanged compared to uninjured corneas (Figure 5B). Since TGFβ/TGFβR signals for the formation of myofibroblasts, this is consistent with the results of the αSMA staining, in which we noted reduced myofibroblasts with 0.1% NLE treatment (Figure 4F). These findings suggest 0.1% NLE suppresses TGFβ/TGFβR signaling, resulting in decreased levels of myofibroblasts. With reduced myofibroblast activity, 0.1% NLE-treated corneas demonstrate less fibrotic healing (Figure 1).

### 2.7. Reverse Transcription PCR Measure of Gene Expression

**Figure 6:**
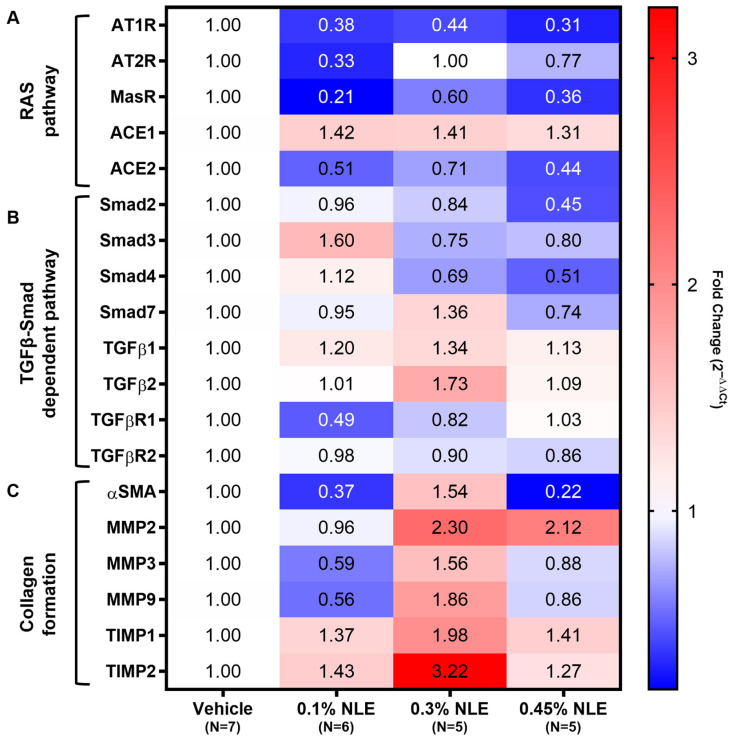
Relative gene expression in injured corneas. Fold change is in comparison to vehicle and expressed as 2^−ΔΔCt^. Targets evaluated include components related to the RAS pathway (**A**), TGFβ-Smad-dependent pathway (**B**), and collagen formation (**C**).

The relative gene expression of various RAS- and fibrosis-related targets was evaluated to investigate the molecular mechanisms by which NLE exerts its biological effect (Figure 6). MasR corneal gene expression trended towardsreduction with NLE treatment, which may reflect a ligand-dependent receptor downregulation following sustained MasR activation or reflect the reduced number of fibrotic cells that express RAS components (Figure 6A). Compensatory downregulation trends of AT1R and AT2R was observed as well. ACE2, which metabolizes Ang(1-8) to form Ang(1-7), trended towards a downregulation of gene expression with NLE treatment (Figure 6A). This is expected given that an exogenous supply of an Ang(1-7) analogue was introduced in these corneas.

The gene expression of components of the Smad pathway was relatively unaffected by 0.1% NLE treatment (Figure 6B). While TGFβ1 trended towards slight upregulation in NLE treatment groups, TGFβR1 showed downregulation (Figure 6B). TGFβR1 downregulation is consistent with the results of the immunofluorescence staining shown in Figure 5C. A reduction trend in αSMA expression was noted in 0.1% NLE- and 0.45% NLE-treated corneas (Figure 6C), consistent with the αSMA levels measured via immunofluorescent staining (Figure 4F) and decreased levels of corneal haze in these treatment groups (Figure 1).

Matrix metalloproteinases (MMPs) are secreted by myofibroblasts to break down damaged collagen in response to injury. MMP2, 3, and 9 gene expression trends showed downregulated with 0.1% NLE, while their natural inhibitors, the tissue inhibitors of matrix metalloproteinase 1 and 2 (TIMP1 and 2) trended towards upregulation, indicating a reduction in collagen degradation (Figure 6C). This is consistent with the increased levels of collagen measured via Masson trichrome staining (Figure 3). Similar but less defined trends in MMP and TIMP expression were observed in the 0.45% NLE treatment group. In contrast, 0.3% NLE-treated corneas show elevated expression of αSMA, MMPs, and TIMPs. This loss of control over MMP/TIMP balance is consistent with an increase in fibrosis.

## 3. Discussion

The 6 mm stellate corneal injury is a full-thickness wound disrupting the EBM and Descemet’s membrane and exposing the stroma to the continuous release of TGFβ1, which triggers the differentiation of keratocytes into myofibroblasts when it binds to TGFβR on the cell surface of keratocytes. Without intervention, these myofibroblasts produce excessive and disorganized ECM that is rich in type III collagen and fibronectin, lacking the keratan–sulfate proteoglycans (lumican and keratocan) that are important in restoring optical transparency [16,24]. Additionally, myofibroblasts themselves are opaque due to the decreased crystallin content [12]. The resulting imbalance between ECM synthesis and degradation, compounded by elevated connective tissue growth factor (CTGF) and MMP/TIMP dysregulation, culminates in stromal fibrosis and permanent light scatter, clinically manifesting as corneal haze. Thus, for clear corneal healing to occur, myofibroblasts must be cleared, either by apoptosis or de-differentiation to keratocytes [25,26,27].

Therapeutic modulation of the fibrotic cascade can be achieved through inhibiting TGFβ1 signaling. Anti-TGFβ1 monoclonal antibodies, small-molecule TGFβR1 kinase inhibitors, or decorin mimetics can sequester TGFβ1 activity [28,29,30,31]. These approaches can attenuate the fibroblasts’ differentiation into myofibroblasts and reduce aberrant ECM deposition [32]. In experimental corneal wound models, the inhibition of TGFβ1 activity during the proliferative phase can preserve keratocyte viability, restore basement membrane integrity, and promote regeneration of the native stromal architecture with markedly reduced haze and fibrosis [28,29,33]. In this study, the downregulation of TGFβR1 was noted. The gene expression data were further supported by IF staining, revealing that while TGFβ1 levels are not significantly affected, TGFβR1 expression was significantly reduced (Figure 5B). These findings suggest that NLE/MasR activation may disrupt TGFβ/TGFβR signaling in the stromal cells (keratocytes and myofibroblasts), which reduces the levels of myofibroblasts expressing αSMA (Figure 4F). This is consistent with reduced MMP and TIMP expression (Figure 6C). Additionally, 0.1% NLE treatment increased keratocyte density, as shown by HRT-III imaging, indicating that NLE can support the restoration of a healthy stromal cell population (Figure 3). In summation, these findings demonstrate MasR activation can accelerate corneal healing while decreasing pro-fibrotic corneal repair, as reflected by the reduction in corneal haze (Figure 1).

The activation of AT1R by angiotensin II (AngII) has known pro-fibrotic effects, including the activation of mitogen-activated protein kinase (MAPK), as well as extracellular signal-regulated kinases 1 and 2 (ERK1/2), promoting fibroblast proliferation and ECM production [34]. MasR activation using Ang(1-7) can counteract this activity, inhibiting MAPK and ERK(1/2) [35]. Additionally, MasR activation by Ang(1-7) has been shown to inhibit the TGFβ-mediated phosphorylation of Smad2 and Smad3 [36]. Similarly, NLE treatment can leads towards downregulation of both AT1R and TGFβR1 expression (Figure 6) through MasR activation. Our findings are consistent with AT1R inhibition via the use of angiotensin receptor blockers (ARBs). Several human case reports and animal studies have suggested that the ARB inhibition of AT1R using losartan is an effective therapeutic strategy to prevent corneal scarring and haze [37,38,39,40]. Topical losartan was shown to be an effective treatment that reduces corneal haze following several types of injuries, including alkali burn injuries, corneal incisions, phototherapeutic keratectomy (PTK), and photorefractive keratectomy (PRK) [37,38,39,40]. Losartan acts via the inhibition of AT1R, blocking phosphorylation of ERK(1/2) and triggering apoptosis of myofibroblasts [41]. Here, we show that 0.1% NLE can also disrupt AngII/AT1R signaling by downregulating the gene expression of AT1R.

The novel 6 mm stellate injury presented in this study is an exceptionally severe injury that better mimics open globe injuries seen in the combat theater than linear lacerations or corneal trephinations. An injury of this size and severity will naturally result in greater variability and increased corneal haze in wound response than a smaller injury. Following an injury this severe, corneal haze will persist for months or years. Therefore, unsurprisingly, no differences in corneal haze were noted in the central cornea by Day 28. An extended study that monitors repair over several months would likely reveal enhanced overall wound resolution. The 0.1% and 0.45% NLE treatments show less variability in multiple important markers of fibrosis, such as corneal haze, edema, and myofibroblasts. Furthermore, 0.1% NLE treatment demonstrated consistent measurements of TGFβR1 and collagen deposition. This phenomenon suggests that NLE may be capable of driving the therapeutic response towards favorable outcomes, even in cases of severe injury that would otherwise result in a highly variable healing response. In contrast, the 0.3% NLE treatment shows similar variability to the vehicle group, suggesting that the wound variability overcomes the therapeutic effect in this group and/or NLE does not have a dose-dependent effect. MasR, as a G-protein coupled receptor (GPCR), is susceptible to receptor desensitization. Ang(1-7) stimulation of MasR causes rapid internalization of the receptor, reducing receptor availability at the cell surface [42]. At high concentrations, Ang(1-7) is known to lead to a diminished response, suggesting receptor desensitization [43,44]. It is likely that the stimulation of MasR by NLE has a similar effect, explaining why the 0.1% NLE dose proved to be most efficacious compared to 0.3% and 0.45% NLE doses.

This study identified 0.1% NLE as the optimal NLE concentration capable of promoting clear corneal healing following a severe, 6 mm full-thickness stellate laceration. We observed that MasR activation by 0.1% NLE exerted anti-fibrotic effects via disruption of the TGFβ/TGFβR signal, though it is unclear how NLE downregulates TGFβR1 protein and gene expression. The 0.1% NLE dose promotes corneal healing while reducing corneal edema and haze (Figure 1 and Figure 2C). These results support that MasR activation can promote anti-fibrotic and anti-inflammatory wound repair, as shown previously in a smaller full-thickness corneal injury [23]. 

One limitation of this study is the exclusion of an injured, untreated or an uninjured, untreated treatment groups. These groups were not included in the study design in order to minimize the number of animals needed to complete the study; however, these groups would likely serve as a better comparator for baseline gene expression and may have provided a clearer picture of the differences between molecular mechanism with NLE administration. Additionally, we elected to focus on the effects of RAS on the TGFβ Smad-dependent pathway, but NLE may act on Smad-independent pathways like ERK(1/2) or inflammatory pathways such as nuclear factor κB (NFκB). Furthermore, the ability of NLE to downregulate AT1R and TGFβR expression, as shown in this study, needs to be further investigated in order to better discern the precise mechanism by which this occurs.

## 4. Materials and Methods

### 4.1. Animal Study Design

All animal experiments were approved by the Institutional Animal Care and Use Committee of University of Southern California and conducted in accordance with the Association for Research in Vision and Ophthalmology (ARVO) Statement for the Use of Animals in Ophthalmic and Vision Research. Thirty (30) New Zealand Black (NZB) rabbits (2–3 kg) were randomly assigned to receive two eye drops (~100 μL) twice daily of one of four treatments (vehicle, 0.1%, 0.3%, or 0.45% NLE). These doses were chosen based on previous studies completed by our group, including the Abdullah et. al. study [23]. Eyedrop application began immediately following surgery and continued at twice daily dosing for the full 28 day study. For each ocular examination, rabbits were anesthetized via intramuscular injection of ketamine hydrochloride (35–50 mg/kg) and xylazine (5–10 mg/kg). Topical anesthesia was provided using tetracaine (oxybuprocaine 0.4%) prior to imaging. Atipamezole (5–10 mg/kg) was administered to reverse anesthesia.

### 4.2. Surgical Procedure

The periorbital area was sterilized with 5% povidone–iodine solution before positioning sterile drapes and an eye speculum. The central cornea was marked in three directions, each measuring 6 mm in length and crossing through the center of the cornea (from 0° to 180°, 60° to 240°, and from 120° to 300°). A diamond blade made partial-thickness incisions along the previously marked directions. An entry point was created at the center of the partial-thickness incisions, and an ophthalmic viscosurgical device (OVD) was injected to reform the anterior chamber. The marked areas of the cornea were cut with scissors, creating a full-thickness wound in a stellate shape. Injury induction was performed by the same surgeon throughout the study, and the injury was positioned and induced consistently for each subject. Buprenorphine SR (0.12–0.15 mg/kg) was administered subcutaneously immediately prior to surgery and as needed for pain management. Antibiotic ointment (neomycin, polymyxin, bacitracin) was administered once daily for 3 days following surgery.

### 4.3. Ocular Examination

Ocular examinations were conducted prior to surgery and at 1, 3, 7, 14, 21, and 28 days post-injury. At ocular examinations, IOP measurements and Seidel’s test results were recorded. Additionally, non-invasive corneal imaging including slit lamp biomicroscopy and corneal confocal microscopy using HRT-III was performed.

### 4.4. Seidel’s Pressure Test

To assess corneal wound leakage, BioGlo™ fluorescein ophthalmic strips (Hub Pharmaceuticals, Rancho Cucamonga, CA, USA) were administered to the conjunctival sack. Under cobalt blue light, any changes in the color or surface of the cornea indicate a corneal leakage and were recorded as a positive Seidel’s test result. No changes to color or surface of the cornea indicate a closed wound and were recorded as a negative Seidel’s test result. Corneas with negative Seidel’s tests at all follow ups were defined as having complete wound closure within 24 h.

### 4.5. Heidelberg Retinal Tomogram (HRT-III)

A Rostock Cornea Module was used with HRT-III (Heidelberg Engineering, Heidelberg, Germany) to capture the confocal microscopy of each cornea at all ocular examinations. Images were captured with dimensions of 384 × 384 pixels, covering 400 × 400 μm. Confocal microscopy images were captured at the edge of the wound at various depths from the epithelial surface.

To agnostically quantify keratocytes, an AI model was trained to count keratocytes based on morphology using the image analysis platform Biodock.ai [45]. Five images were randomly selected from the stroma for each cornea. The keratocyte count from the five fields was averaged to obtain the count for each cornea.

To evaluate the extent of corneal edema, the thickness of the stroma at Day 0 and Day 28 was measured by three blinded evaluators using the depths recorded on each HRT-III image. The anterior-most stromal image was compared to the posterior-most stromal image to determine the thickness of the stroma. The change in stromal thickness between Day 28 and Day 0 was evaluated as a surrogate marker of corneal edema.

### 4.6. Corneal Haze Measurement

Slit lamp images from each timepoint were assessed for corneal haze. Three masked graders evaluated images of each cornea and defined haze as none (0), trace (0.5), minimal (1), mild (2), moderate (3) or severe (4) based on a previously defined haze grading scale (Table S1) [46]. The graders assigned a haze score to both the central cornea at the injury site and the peripheral cornea, near the limbus. The median scores for each subject and timepoint were calculated.

In addition, the area of corneal haze was quantified. In ImageJ, the area in pixels of corneal haze was measured and normalized to the total area of the cornea in pixels to obtain a percent area of corneal haze.

### 4.7. Euthanasia and Tissue Collection

At the end of study, rabbits were euthanized by intravenous injection of pentobarbital sodium and phenytoin sodium (Euthasol^®^, Fort Worth, TX, USA). Eyes were enucleated and dissected to isolate the cornea and aqueous humor. The cornea was cut in half at the center of the wound. One half was immersed in Davidson’s fixative solution for histological analysis while the other half was flash frozen for gene expression analysis.

### 4.8. Masson’s Trichrome

Corneas were removed from Davidson’s fixative after 24 h and placed in 70% ethanol and transferred to USC Ginsburg Institute for Biomedical Therapeutics core for paraffin embedding and sectioning. Serial cross-sections were taken starting at the center of the wound, moving towards the limbus. Corneal sections were deparaffinized and rehydrated via passage through a series of xylene, ethanol, and PBS. Corneas were stained via Masson’s trichrome using Trichome Stain Kit (abcam, Cambridge, UK). Images at 40× magnification were captured using an Olympus BX43 microscope. Collagen content was measured using the color deconvolution function in ImageJ and normalized to tissue area to produce a percent area of collagen.

### 4.9. Immunofluorescence Staining of αSMA

Corneal sections were placed in a heated antigen retrieval buffer (80 °C) for 30 min then blocked with 0.1% Triton-X and 2.5% normal goat serum in PBS for 30 min at room temperature. Sections were incubated with mouse anti-αSMA monoclonal antibody (Abcam, Cambridge, UK) diluted 1:500 with blocking buffer at 4 °C overnight, and then incubated with goat anti-mouse Alexa Fluor™ 594 (ThermoFisher Scientific, Waltham, MA, USA) diluted 1:500 with blocking buffer for 45 min at room temperature. Coverslips were mounted using VECTASHIELD Vibrance Antifade Mounting Medium with DAPI (Vector Laboratories, Newark, CA, USA). Ten images at 40× magnification were captured throughout the stroma using an Olympus BX43 microscope. Area of αSMA stain in pixels was quantified using ImageJ at a manually set threshold of (20, 255). The area of αSMA stain for each set of fields was averaged.

### 4.10. Immunofluorescence Staining of TGFβ1 and TGFβR1

Corneal sections were placed in a heated antigen retrieval buffer (80 °C) then incubated with blocking buffer (0.1% Triton-X and 2.5% normal donkey serum in PBS) for 30 min at room temperature. Sections were incubated with mouse anti-TGFβ1 monoclonal antibody (Abcam, Cambridge, UK) diluted 1:500 with blocking buffer at 4 °C overnight and then with goat anti-mouse Alexa Fluor™ 488 (ThermoFisher Scientific, Waltham, MA, USA) diluted 1:500 with blocking buffer for 45 min at room temperature. Sections were then placed in blocking buffer for 30 min at room temperature. Sections were incubated with goat anti-TGFβR1 monoclonal antibody (Abcam, Cambridge, UK) diluted 1:500 with blocking buffer at 4 °C overnight and then with donkey anti-goat Alexa Fluor™ 594 for 45 min at room temperature. Slides were counterstained with DAPI at 1 μg/mL for 10 min and then mounted with VECTASHEILD Vibrance Antifade Mounting Medium (Vector Laboratories, Newark, CA, USA). Five images at 40× magnification were captured in the stroma using an Olympus BX43 microscope. The area of TGFβ1 and TGFβR1 stain in pixels was quantified and averaged using ImageJ (version 1.54g) at manually set thresholds of (17, 255) and (15, 255), respectively.

### 4.11. Reverse-Transcription PCR

Total RNA was extracted from homogenized corneas using RNAzol RT (Sigma-Aldrich, St. Louis, MO, USA) following the manufacturer’s protocol. RNA concentration and purity were assessed using NanoDrop™ One (ThermoFisher Scientific, Waltham, MA, USA). Reverse transcription was performed using RevertAid RT kit (ThermoFisher Scientific, Waltham, MA, USA). Rabbit primers were designed using NCBI primer blast (Table S2). Samples were preamplified with SsoAdvanced PreAmp Supermix (Bio-Rad, Hercules, CA, USA) following manufacturer’s guidelines. Each RT-PCR reaction included 10 ng preamped cDNA, 300 nM forward and reverse primers, and SYBR Green Master Mix (ThermoFisher Scientific, Waltham, MA, USA) on a 384 well plate. RT-PCR was performed on Applied Biosystems QuantStudio 12K Flex Real-Time PCR System (ThermoFisher Scientific, Waltham, MA, USA). Data was analyzed using the 2^−ΔΔCt^ method with β-actin and h18s as reference genes.

### 4.12. Statistical Analysis

All statistical analyses were performed using GraphPad Prism version 8.4.3 (GraphPad Prism Software, La Jolla, CA, USA). A one-way ANOVA and Tukey’s multiple comparisons test were used on datasets with a Gaussian distribution. Kruskal–Wallis and Dunn’s multiple comparisons tests were used on discrete or nonnormal datasets. Statistical significance was determined using an α of 0.05.

## 5. Conclusions

This study identified 0.1% NLE as the optimal dose to promote minimally fibrotic corneal healing following severe stellate corneal injury. Molecular interrogation revealed that NLE exerts its biological effect in part through the blockage of AT1R and TGFβ1R activation. Treatment with 0.1% NLE led to increased corneal deposition, reduced corneal haze, and improved corneal organization. Immunofluorescence staining of TGFβR1 and αSMA suggests modulation of corneal cellular biology. Additional studies will be necessary to determine the molecular sequence and show how NLE/MasR can affect AT1R and TGFβR signaling in relation to corneal repair mechanisms.

## Data Availability

The original contributions presented in this study are included in the article/Supplementary Material. Further inquiries can be directed to the corresponding author.

## Supplementary Materials

The following supporting information can be downloaded at: https://www.mdpi.com/article/10.3390/ijms27083565/s1.

## Abbreviations

The following abbreviations are used in this manuscript:

ECM	Extracellular matrix
TGFβ	Transforming growth factor beta
NLE	NorLeu^3^Angiotensin(1-7)
RAS	Renin–angiotensin system
NZB	New Zealand Black
αSMA	A-smooth muscle actin
IF	Immunofluorescence
EBM	Epithelial basement membrane
TGFβR	Transforming growth factor beta receptor
AT1R	Angiotensin type 1 receptor
IOP	Intraocular pressure
AI	Artificial intelligence
MMP	Matrix metalloproteinase
TIMP	Tissue inhibitor of matrix metalloproteinase
CTGF	Connective tissue growth factor
AngII	Angiotensin II
MAPK	Mitogen activated protein kinase
ERK	Extracellular signal-regulated kinases 1 and 2
ARB	Angiotensin receptor blockers
PTK	Phototherapeutic keratectomy
PRK	Photorefractive keratectomy
ARVO	Association for Research in Vision and Ophthalmology
OVD	Ophthalmic viscosurgical device
