# Supplementary material for: Administration of Topical NorLeu3Angiotensin(1-7) Minimizes Fibrotic Corneal Healing in Stellate Wound: A 28-Day Study
Source: Int J Mol Sci. 2026 Apr 16;27(8):3565. doi: 10.3390/ijms27083565 (PMC13115795; doi:10.3390/ijms27083565)
Supplement: Supplementary file 1 [file ijms-27-03565-s001.zip › Supplementary Figures Captions.pdf]

Supplementary Figure S1. Average IOP over time.

Average IOP (mm Hg) of each treatment group throughout the study. Dotted lines identify normal IOP range (7-15 mm Hg).

Supplementary Figure S2. Corneal haze over time.

A. Area of corneal haze in pixels, normalized to corneal area in pixels. B. Average area haze for each treatment group is plotted, with error bars representing SD.

Supplementary Figure S3. Keratocyte density throughout study.

A. Keratocyte density measured at each timepoint. Average keratocyte density at baseline is 77.4 keratocytes/400 μm^2 (dotted line). B. Average keratocyte density for each treatment group is plotted, with error bars representing SD.
