# Supplementary material for: Administration of Topical NorLeu3Angiotensin(1-7) Minimizes Fibrotic Corneal Healing in Stellate Wound: A 28-Day Study
Source: Int J Mol Sci. 2026 Apr 16;27(8):3565. doi: 10.3390/ijms27083565 (PMC13115795; doi:10.3390/ijms27083565)
Supplement: Supplementary file 1 [file ijms-27-03565-s001.zip › Supplementary Tables.pdf]

Corneal Haze Grading Criteria	
Score	Criteria
0	Clear cornea, no opacity seen.
0.5	Trace haze, barely visible.
1	Minimal haze, only seen with close examination.
2	Mild haze, easily visible.
3	Moderate haze, obstructs details of iris.
4	Severe opacity, completely obstructs iris and other intraocular structures.

Supplementary Table S1. Haze grading criteria, adapted from Fantes et. al.

Primer Sequences used for Reverse Transcription (RT) PCR		
**Gene**	**Forward Primer Sequence**	**Reverse Primer Sequence**
β Actin	CAGAAGGACTCGTACGTGGG	CATGTCGTCCCAGTTGGTCA
h18s	AAACGGCTACCACATCCAAG	CAATTACAGGGCCTCGAAAG
AT1R	GCCCTCAAGAAGGCTTACGA	TCTGCAGTCGTGAATGACCC
AT2R	CCTTGTGTGGTGTATGGCCT	GCTATCCCAACCGACCACTG
MasR	GGGGACTCCACTGATTGGTT	TTGGGGCAGTGGTCTCATTG
ACE1	CTCCCCCAGAAAGAGGCTTG	ATGGCAGGACGATGGTCAGT
ACE2	GCATGTCCCGGAGTCGTATC	CAGCCATATGGGGACAGGTG
Smad2	CAGGCCTTTACAGCTTCTCTGA	CCTTTCCGATGGGACACCTG
Smad3	GCTGGACGACTACAGCCATT	TGTGGTTCATCTGGTGGTCG
Smad4	CGCGGATCAACCGAGACATA	TACTGGCAGGCTGACTTGTG
Smad7	CAGGCATTCCTCGGAAGTCA	CACCAGTTCGGGGTTGATCT
TGFβ1	ACATCTACACAGTTCCCGGC	TCCAGGCTCCAGATGTAGGG
TGFβ2	CGAGGAGTACTACGCCAAGG	AACTGGGCAGACAGTTTCGG
TGFβR1	GGACCAGTCTGCTTCGTCTG	TGATCCCGAACCCGATGTTG
TGFβR2	CCCCCGCACGTTCATAAGT	GTCACAGGTGGAAGATCGCA
αSMA	AAGGAAATCACGGCCCTAGC	CGGAGTATTTGCGCTCTGGA
MMP2	GCTGCGGTTTTCTCGAATCC	TATCCGTCTCCATGCTCCCA
MMP3	TCAAGGGATGCAGACACCAC	TCACCTCCAAGCCAAGGAAC
MMP9	GGGTATCCTTTCGACGGCAA	GTCGGCGTTTCCAAAGTACG
TIMP1	TCTCTCAACGTTCCGGCTTC	GGATGCACAGGCAAACACTG
TIMP2	TGAGAGTCGTTGAGGGTCCA	ATTGTCCATTGACCGAGCGA

Supplementary Table S2. RT-PCR primer sequences. Rabbit primers were designed using the NCBI Blast Tool and purchased from Integrated DNA Technologies (IDT).
