# Supplementary figures and images for: Administration of Topical NorLeu3Angiotensin(1-7) Minimizes Fibrotic Corneal Healing in Stellate Wound: A 28-Day Study
Source: Int J Mol Sci. 2026 Apr 16;27(8):3565. doi: 10.3390/ijms27083565 (PMC13115795; doi:10.3390/ijms27083565)

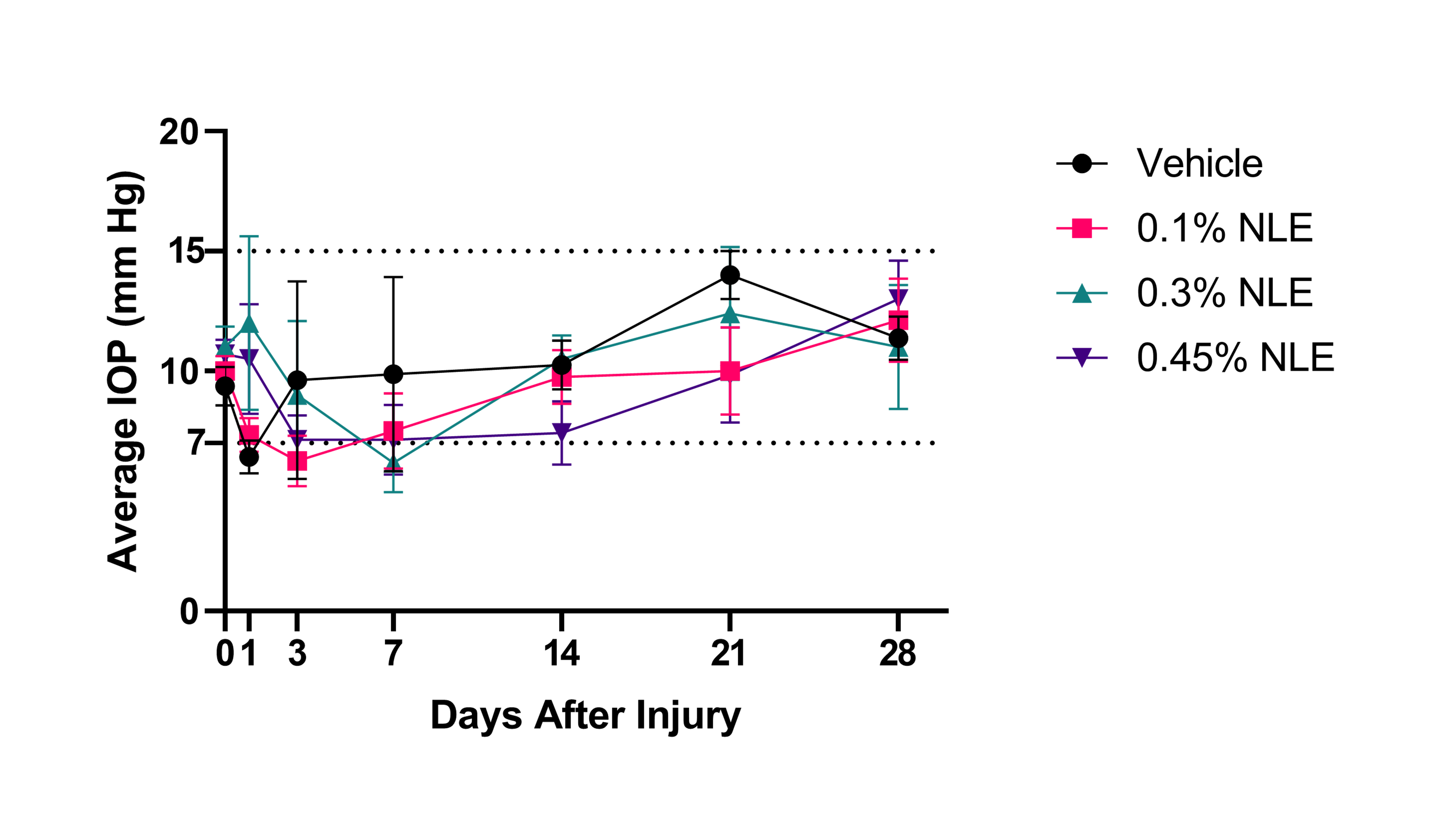

Supplement: Supplementary file 1 [file ijms-27-03565-s001.zip › Figure S1.tif]

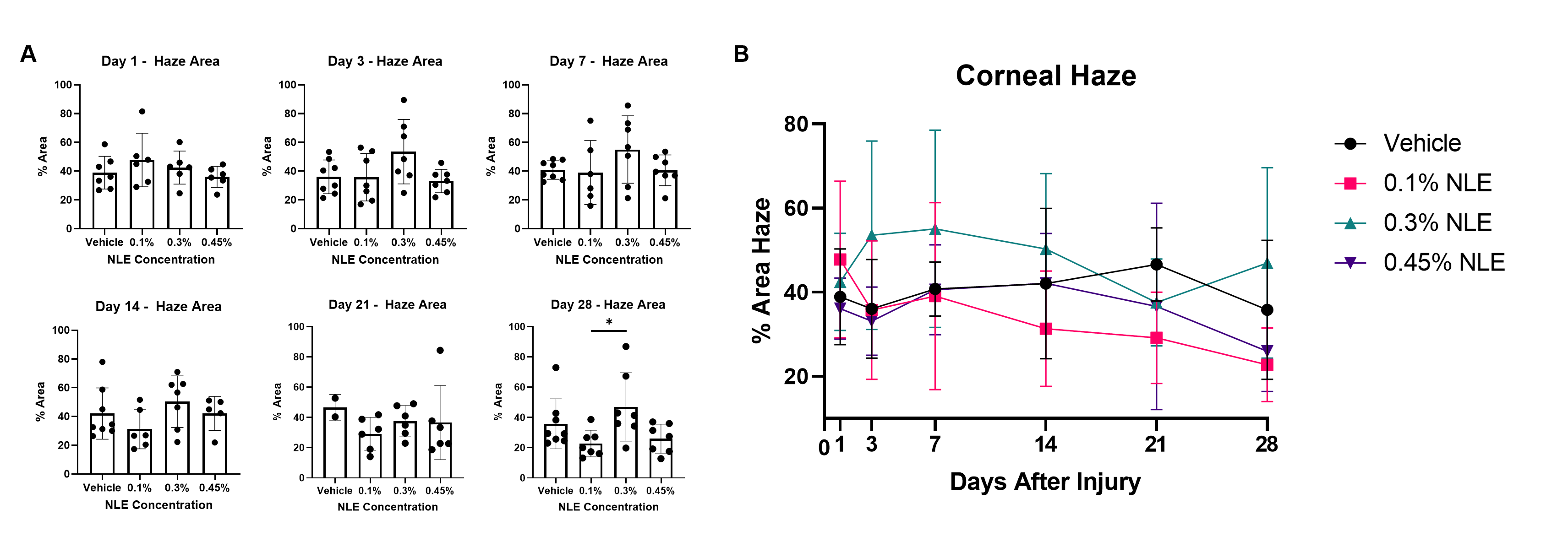

Supplement: Supplementary file 1 [file ijms-27-03565-s001.zip › Figure S2.png]

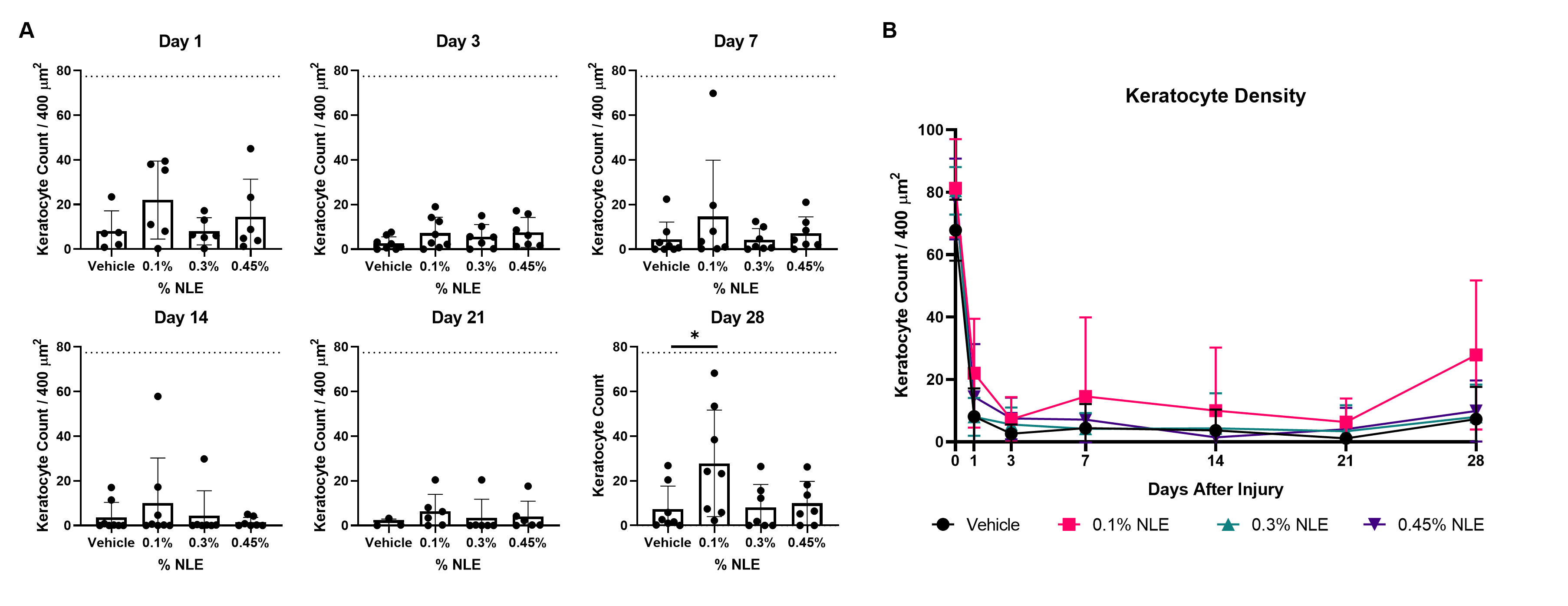

Supplement: Supplementary file 1 [file ijms-27-03565-s001.zip › Figure S3.png]
